# 2B and 3C Proteins of Senecavirus A Antagonize the Antiviral Activity of DDX21 *via* the Caspase-Dependent Degradation of DDX21

**DOI:** 10.3389/fimmu.2022.951984

**Published:** 2022-07-14

**Authors:** Kuan Zhao, Xiao-Ran Guo, Shuai-Feng Liu, Xiao-Na Liu, Ying Han, Lu-Lu Wang, Bai-Shi Lei, Wu-Chao Zhang, Li-Min Li, Wan-Zhe Yuan

**Affiliations:** ^1^ College of Veterinary Medicine, Hebei Agricultural University, Baoding, China; ^2^ Hebei Veterinary Biotechnology Innovation Center, Hebei Agricultural University, Baoding, China; ^3^ North China Research Center of Animal Epidemic Pathogen Biology, China Agriculture Ministry, Baoding, China

**Keywords:** senecavirus A (SVA), DDX21, 2B protein, 3C protein, caspase pathway

## Abstract

Senecavirus A (SVA), also known as Seneca Valley virus, is a recently discovered picornavirus that can cause swine vesicular disease, posing a great threat to the global swine industry. It can replicate efficiently in cells, but the molecular mechanism remains poorly understood. This study determined the host’s differentially expressed proteins (DEPs) during SVA infection using dimethyl labeling based on quantitative proteomics. Among the DE proteins, DDX21, a member of the DEAD (Asp-Glu-Ala-Asp)-box RNA helicase (DDX) family, was downregulated and demonstrated inhibiting SVA replication by overexpression and knockdown experiment. To antagonize this antiviral effect of DDX21, SVA infection induces the degradation of DDX21 by 2B and 3C proteins. The Co-IP results showed that 2B and 3C did not interact with DDX21, suggesting that the degradation of DDX21 did not depend on their interaction. Moreover, the 3C protein protease activity was necessary for the degradation of DDX21. Furthermore, our study revealed that the degradation of DDX21 by 2B and 3C proteins of SVA was achieved through the caspase pathway. These findings suggest that DDX21 was an effective antiviral factor for suppressing SVA infection and that SVA antagonized its antiviral effect by degrading DDX21, which will be useful to guide further studies into the mechanism of mutual regulation between SVA and the host.

## Introduction

Senecavirus A (SVA), a nonenveloped single-strand positive-sense RNA virus, is the only member of the genus *Senecavirus* within the family *Picornaviridae.* As a serendipitous finding, it was first discovered in the cell line PER.C6 cultivating adenovirus-5-based vectors in 2002 (1). The genome of SVA is about 7.3 kb and encodes only one polyprotein, which follows the standard L-4-3-4 layout for picornavirus genomes. The polyprotein is processed into the structural and nonstructural proteins by proteases 2A and 3C, including the leader protein and three major protein regions (P1, P2, and P3) (2). Until 2014, only three complete genomic sequences of SVA were available in the National Center for Biotechnology Information databases (GenBank accession numbers NC_011349, DQ641257, and KC667560), and the biological properties and the pathogenicity of SVA for swine were unknown. In early 2015, numerous SVA strains were isolated and reported in vesicular disease outbreaks in Brazil, China, and Thailand (3). The diseased swine are characterized by severe vesicular and/or ulcerative lesions on the oral mucosa, snout, coronary bands, and hooves, which are indistinguishable from the clinical symptoms caused by the foot-and-mouth disease virus (FMDV) and vesicular stomatitis virus (VSV). From then, SVA was confirmed as the agent of the vesicular disease of swine and began to spread in many countries.

The hosts have developed highly efficient strategies to detect and control invading viruses to resist infection and maintain a normal physiological state. In contrast, most viruses have evolved strategies to evade host defenses and thus effectively infect and replicate in host cells. Increasing evidence suggests that SVA can escape the host’s antiviral effect in several ways for better infection and replication. For example, the 2B protein of SVA, whose secondary structures are similar to those of the picornaviruses, can act like a viroporin and likely enhance membrane permeability. It interacted with mitochondrial antiviral signaling (MAVS) and induced the degradation of MAVS depending on caspase-9 and caspase-3 to suppress the activation of the RLR pathway (4). Furthermore, the 3C protein of SVA inhibited antiviral type I IFN responses by targeting different host adaptors, including MAVS, Toll/interleukin 1 (IL-1) receptor domain-containing adaptor inducing IFN-β (TRIF), and TRAF family member-associated NF-κB activator (TANK).

Moreover, the SVA 3C protein reduces interferon regulatory factor 3 (IRF3) and IRF7 protein expression levels and phosphorylation and blocks the transcription of IFN-β, IFN-α1, IFN-α4, and ISG54 (5). More and more evidence indicated that 3C protein plays a crucial role in modulating virus and host gene expression by cleaving and degrading various host protein factors. Furthermore, the protease activity of 3C protein is required for these reactions (6). Therefore, the relationship between virus and host should be investigated to uncover the mechanisms of SVA antagonizing the host antiviral effect.

DEAD (Asp-Glu-Ala-Asp)-box RNA helicases (DDXs) are the largest family of evolutionarily conserved RNA helicases that are involved in a broad array of host processes, especially in antiviral immunity (7–9). DDX21, a member of the DDX family, possesses all the signature motifs required for DEAD-helicase function and contains atypical FRGQR repeats in its C-terminus. Furthermore, growing evidence suggests that DDX21 plays an important role in regulating host antiviral immunity. For example, DDX21 inhibits influenza viral RNA synthesis by binding to the PB1 polymerase subunit (10). Furthermore, DDX21 regulated the replication of FMDV by increasing IFN-β and IL-8 production in FMDV-infected cells. It also coprecipitates with FMDV IRES and restricts viral IRES-dependent translation and replication (11). Moreover, the infection process of human cytomegalovirus, human immunodeficiency virus, and dengue virus were also regulated by DDX21. However, the reciprocal regulation mechanism between DDX21 and SVA is still unclear.

Our study has determined changes in the host cell protein expression during SVA infection using dimethyl labeling-based quantitative proteomics. Among these proteins, the expression level of DDX21 was significantly downregulated during SVA infection, and the antiviral effect of DDX21 was also verified by overexpressing and knockdown experiments. Moreover, SVA 2B and 3C proteins induce the degradation of DDX21 *via* caspase to antagonize the antiviral activity of DDX21. The results helped study the interaction between host and virus.

## Materials and Methods

### Cells, Viruses, and Drug

Porcine kidney-15 (PK-15), human embryonic kidney (HEK293T) cells, and baby hamster kidney cells (BHK-21) cells were cultured in Dulbecco’s modified Eagle’s medium containing 10% fetal bovine serum (Gibco, Thermo Fisher Scientific, Waltham, MA, USA). Cells were maintained at 37°C with 5% CO_2_. The SVA virus (GenBank accession number: MZ375462) was used for all the experiments. The proteasome inhibitor MG-132, the lysosomal inhibitor chloroquine (CQ), and the caspase inhibitor carbobenzoxy-valyl-alanyl-aspartyl-[*O*-methyl]-fluoro-methyl ketone (Z-VAD-FMK) was purchased from Beijing Solarbio Science & Technology Co. Ltd.

### Construction of Plasmids

The porcine DDX21 gene (GenBank accession number: XM_005657387.3) was amplified and cloned into pCAGEN (modified from pCAGGS) to generate pCAGEN-DDX21-HA. The other proteins of SVA were cloned into pCAGGS to generate pCAGGS-VP1-Flag, pCAGGS-VP2-Flag, pCAGGS-VP3-Flag, pCAGGS-2B-Flag, pCAGGS-2C-Flag, pCAGGS-3A-Flag, pCAGGS-3C-Flag, and pCAGGS-3D-Flag. All plasmids were constructed by homologous recombination with the NEBuilder^®^ HiFi DNA Assembly Master Mix (New England Biolabs, Ipswich, MA, USA) according to the manufacturer’s instructions. Mutagenesis of SVA 3C protein was generated using overlap PCR and cloned into vector pCAGGS-3C-Flag (H48A), pCAGGS-3C-Flag (C160A, USA), and pCAGGS-3C-Flag (double-site mutation (DM) H48A-C160A). The primers used for gene amplification are listed in **Table 1**.

**Table 1:** The sequence of primers and siRNAs used in the study.

Primers and siRNAs	Sequence (5′-3′)
pCAGGS-VP1-F	CATCATTTTGGCAAAGATGTCCACCGACAACGCCGAGAC
pCAGGS-VP1-FLAG-R	CGAGAGATCTGAATTTCACTTATCGTCGTCATCCTTGTAATCTTGCATCAGCATCTTCTGCT
pCAGGS-VP2-F	CATCATTTTGGCAAAGATGGATCACAATACCGAAGAAAT
pCAGGS-VP2-FLAG-R	CGAGAGATCTGAATTTCACTTATCGTCGTCATCCTTGTAATCCTGTTCCTCGTCCGTCCCGG
pCAGGS-VP3-F	CATCATTTTGGCAAAGATGGGGCCCATTCCCACAGCACC
pCAGGS-VP3-FLAG-R	CGAGAGATCTGAATTTCACTTATCGTCGTCATCCTTGTAATCGTGGAACACGTAGGAAGGAT
pCAGGS-2B-F	CATCATTTTGGCAAAGATGCCTGCTTCTGACAACCCAAT
pCAGGS-2B-FLAG-R	CGAGAGATCTGAATTTCACTTATCGTCGTCATCCTTGTAATCTTGCATCTTAAACAGCTTTC
pCAGGS-2C-F	CATCATTTTGGCAAAGATGGGACCCATGGACACAGTCAAAGA
pCAGGS-2C-FLAG-R	CGAGAGATCTGAATTTCACTTATCGTCGTCATCCTTGTAATCCTGTAGAACCAGAGTCTGCATAT
pCAGGS-3A-F	CATCATTTTGGCAAAGATGAGCCCTAACGAGAACGACGA
pCAGGS-3A-FLAG-R	CGAGAGATCTGAATTTCACTTATCGTCGTCATCCTTGTAATCCTCGCTCCTAGGCGCTTTAG
pCAGGS-3C-F	CATCATTTTGGCAAAGATGCAGCCCAACGTGGACATGGG
pCAGGS-3C-FLAG-R	CGAGAGATCTGAATTTCACTTATCGTCGTCATCCTTGTAATCTTGCATTGTAGCCAGAGGCT
pCAGGS-3D-F	CATCATTTTGGCAAAGATGGGACTGATGACCGAGCTAGAGCCT
pCAGGS-3D-FLAG-R	CGAGAGATCTGAATTTCACTTATCGTCGTCATCCTTGTAATCGTCGAACAAGGCCCTCCATCTTGCC
pCAGGS-3C-H48A-F	CCTTCCTAGTCAATGAGGCCACATGGTCCAACCCCTCCTG
pCAGGS-3C-H48A-R	CAGGAGGGGTTGGACCATGTGGCCTCATTGACTAGGAAGG
pCAGGS-3C-C160A-F	GTGACGACTTACAAGGGATGGGCCGGTTCGGCCCTGGTCTGTGA
pCAGGS-3C-C160A-R	TCACAGACCAGGGCCGAACCGGCCCATCCCTTGTAAGTCGTCAC
pCAGEN-DDX21-HA-F	GTTCCAGATTACGCTGAAATGCCGGGGAAACTTCGTAGT
pCAGEN-DDX21-R	GGGTACCATCGATGAATTACTGTCCAAACGCTTTGCTAAA
Q-STAT1-F	CCTGTTGCGGTTCAGTGAGAGC
Q-STAT1-R	GAAGTAAGGTTCGCCTCCGTTCTG
Q-OAS1-F	ACCTCGTCGTCTTCCTCACCAAG
Q-OAS1-R	CCTGTCGTGGAGTCTGGACCTC
Q-MX2-F	CGGCTGTTTACCAAGATGCGAAATG
Q-MX2-R	ATTCACAAACCCTGGCAACTCTCTC
Q-ZAP-F	AGCAGACACCGCCGAAATAAACC
Q-ZAP-R	CAGCAGCCGAAGCAGATGGAAG
Q-IFIT1-F	AGATGGACTGTGAGGAAGGATGGG
Q-IFIT1-R	GGTCTAGGCGATAGATGGTGATTGC
Q-ZC3H11A-F	TCCTCCATTGAAACGTAGCCTTGC
Q-ZC3H11A-R	GCTGACACACCTAATCGCTCCTTC
Q-DDX21-F	CGGACAGGAACTGGGAAGACATTC
Q-DDX21-R	TCTGCTTACTTGACTTGCCAACTCC
Q-IL-18-F	AGACCTGGAATCGGATTACTTTGGC
Q-IL-18-R	ACGGCTTGATGTCCCTGGTTAATG
Q-ZCCHC9-F	ATCAAGTGCAAGGCTCAAGTAGACC
Q-ZCCHC9-R	GCAACCACCATCAGCATAGAGTCC
DDX21-1651-senece	GCAGGAUGCCCAGUCUUUATT
DDX21-1651-antisenece	UAAAGACUGGGCAUCCUGCTT
DDX21-673-senece	GGAAGCCAAUGGAGACAUUT
DDX21-673-antisenece	AAUGUCUCCAUUGGCUUCCTT
DDX21-1712-senece	GGUUUCCGAAAUGGCAGUUTT
DDX21-1712-antisenece	AACUGCCAUUUCGGAAACCTT

### Dynamics of SVA in PK-15 Cells

PK-15 cells were infected with SVA at a multiplicity of infection (MOI) of 0.5, and normal cells were set as the control. Cytopathic effects (CPEs) were observed at 12, 24, 36, 48, 60, and 72 h postinfection (hpi) under a microscope. Subsequently, the cell culture supernatant at different time points was collected to determine 50% tissue culture infective doses (TCID_50_). The viral titers were determined by the Reed–Muench method and expressed as TCID_50_/ml. Mean values and standard deviations were calculated from the results of three independent experiments. According to the flow chart, the cells infected or uninfected with SVA at 48 hpi were chosen for the following quantitative proteomics.

### Sample Preparation, Digestion, and TMT Labeling

Mock- or SVA-infected PK-15 cells were cultured in 6-well plates for 48 hpi. The cells were then washed with cold phosphate-buffered saline (PBS), lysed by sonication in STD lysate buffer [4% (w/v) SDS, 100 mM Tris/HCl pH 7.6, 0.1 M DTT] (12), and quantified using a BCA assay. 200 ug of protein per sample for trypsin digestion using filter-aided sample preparation (FASP) (12), after trypsin digestion, the peptides were desalted on a C18 column and 40 μl of dissolution buffer was added after the lyophilization of peptides. The peptides were quantified by OD280. According to the manufacturer’s instructions, peptides were labeled using a TMT Reagent kit.

### LC-MS/MS Analysis

Labeled samples were analyzed using an Easy-nLC 1200 nanoflow HPLC system (Thermo Fisher Scientific, Waltham, MA, USA). Buffer: solution A is 0.1% formic acid in the water, and solution B is 0.1% formic acid in acetonitrile in water (84% in acetonitrile). The sample was loaded by the autosampler onto the loading column (Thermo Scientific EASY column, 100 μm * 2 cm, 5 μm, C18) and then separated by an analytical column (Thermo Scientific EASY column, 75 μm * 10 cm, 3 μm, C18) with a flow rate of 250 nl/min. After chromatographic separation, the samples were analyzed by a Q Exactive mass spectrometer (13).

### Quantitative Real-Time PCR

Total RNA was extracted with TRIzol (Thermo Fisher Scientific, Waltham, MA, USA). The PrimeScript™ 1st-Strand cDNA Synthesis Kit (TaKaRa, Dalian, China) was used for reverse transcription. The SYBR Premix Ex Taq™ (TaKaRa, Dalian, China) was used to quantify the mRNA levels of ZC3H11A, DDX21, IL-18, ZCCHC9, STAT1, OAS1, MX2, ZAP, and IFIT1. Relative expression levels were analyzed using the 2^−ΔΔCt^ method, with glyceraldehyde-3-phosphate dehydrogenase (GAPDH) mRNA as a control. Primers are listed in **Table 1**.

### Plasmid Transfection and Virus Challenge

To investigate the effect of DDX21 on SVA replication, BHK-21 cells cultured in 6-well plates were transfected with 2 μg of pCAGEN-DDX21-HA using X-treme GENE HP DNA reagent (Roche Applied Science, Penzberg, Germany). Next, 36 h posttransfection (hpt), the cells were infected with SVA at a MOI of 0.1. After inoculation for 1.5 h at 37°C, the supernatants were discarded, and the cells were washed three times with PBS. The supernatant was harvested at 12, 24, 36, and 48 hpi, and the cells were lysed using RIPA lysis buffer (Thermo Fisher Scientific, Waltham, MA, USA). Viral titers in the supernatants were determined using a microtitration assay. The amount of the VP2 protein was then detected in cell lysates by Western blotting (WB) using a rabbit anti-VP2 polyclonal antibody (1:1,000).

### Western Blotting

Cell lysates were prepared by harvesting virus-infected or plasmid-transfected cells in RIPA or IP-lysate buffer at 4°C for 15 min containing 1 mM of phenylmethylsulfonyl fluoride and 1 mg/ml of protease inhibitor cocktail (Roche). After centrifuging at 12,000 rpm/min for 10 min, the supernatants of cell lysates were mixed with 5× sodium dodecyl sulfate-polyacrylamide gel electrophoresis (SDS-PAGE) sample loading buffer (Beyotime) and placed in boiling water for 5 min. The proteins were then separated by SDS-PAGE and transfected onto a nitrocellulose membrane. The membrane was blocked in 5% skim milk for 2 h at room temperature and then incubated with the indicated primary antibody for 1 h at room temperature. After washing three times with Tris-buffered saline with 0.1% Tween 20, the membrane was incubated with horseradish peroxidase-conjugated goat anti-mouse IgG (H+L) and/or goat anti-rabbit IgG (H+L) secondary antibody (1:5,000) for 1 h at room temperature. The target proteins were visualized by treating the membrane with Pierce ECL WB substrate (Thermo Fisher Scientific, Waltham, MA, USA). To quantify target proteins, their levels were normalized to the levels of β-actin.

### RNA Interference

Three small interfering RNAs (siRNAs) against hamster DDX21 (GenBank No. XM_013113937.3) were synthesized by GenePharma (Shanghai, China). The sequence of siRNA is listed in **Table 1**. BHK-21 cells were seeded in 6-well plates and transfected with 100 pM of indicated siRNA or NC using Lipofectamine™ RNAiMAX transfection reagent (cat. No. 13778075; Thermo Fisher Scientific, Waltham, MA, USA). The cells were lysed in RIPA lysis buffer after 36 h of transfection, and the effects of siRNAs were analyzed by WB using an anti-DDX21 polyclonal antibody (cat. No. 10528-1-AP; Proteintech; 1:1,000). Next, efficient siRNA and NC were selected for transfection. At 36 hpt, the cells were infected with SVA at a MOI of 0.1. The supernatant and cells were harvested at 12, 24, 36, and 48 hpi and analyzed based on virus titers and WB.

### Coimmunoprecipitation

HEK293T cells were cotransfected with 1.5 μg/well of DDX21-HA and 1.5 μg/well of 2B-Flag or 3C-Flag using X-tremeGENE DNA transfection reagent. At 24 hpt, cells were lysed with IP lysis buffer containing phenylmethanesulfonyl fluoride and a protease inhibitor cocktail at 4°C for 20 min. Approximately 10% of the lysate supernatant was used as an input control, and the remaining lysates were incubated with anti-Flag agarose beads for 8 h at 4°C. The beads were washed with the lysis buffer five times and then analyzed by WB with the indicated antibodies.

### Cell Viability Assay

Cells at 10^4^ were seeded into 96-well plates and treated with the proteasome inhibitor MG132 concentrations (0–50 μM), the caspase inhibitor Z-VAD-FMK (0–50 μM), or the lysosomal inhibitor CQ (0–100 μM) for 24 h, at five replicate wells per dilution. Subsequently, the 3-(4,5-dimethylthiazol-2-yl)-diphenyl tetrazolium bromide (MTT) assay (14) was used to detect cytotoxicity.

### Statistical Analysis

All the experiments mentioned above were performed with three independent experiments. The GraphPad Prism software was used for statistical analysis by a two-tailed Student’s *t*-test. The data shown are three independent experiments’ means ± standard variations (SD).

## Results

### Virus Infection and Determination of Optimal Collection Time

**Figure 1:**
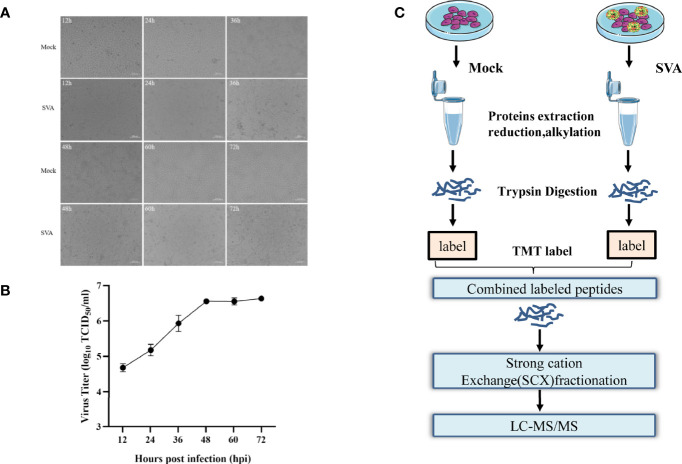
Experimental design for identifying candidate novel cellular factors in mock- and SVA-infected PK-15 cells. **(A)** PK-15 cells were infected with 0.5 MOI of SVA for 12, 24, 36, 48, 60, or 72 h. The virus-induced CPE was observed and recorded by the microscope. **(B)** PK-15 cells were infected with 0.5 MOI of SVA for 12, 24, 36, 48, 60, or 72 h, and the viral titers were determined by the TCID_50_ assay. **(C)** Schematic illustration of the sample preparation. PK-15 cells mock infected or infected by SVA at an MOI of 0.5 for 48 h were subjected to TMT-based proteomic analysis.

PK-15 cells were infected with SVA at a MOI of 0.5, and the CPE began to appear at 36 hpi. Subsequently, the cell showed typical CPE, characterized as rounding, shrinkage, and detachment **(** **Figure 1A** **)**. The virus titers of SVA almost reached the highest value at 48 hpi **(** **Figure 1B** **)**. At 48 hpi, the CPE of the cells was typical, and the virus titers reached the peak, so the cells were collected and subjected to proteomics analyses by LC-MS/MS **(** **Figure 1C** **).**

### Proteomics Analysis of Host Proteins Upon SVA Infection

**Figure 2:**
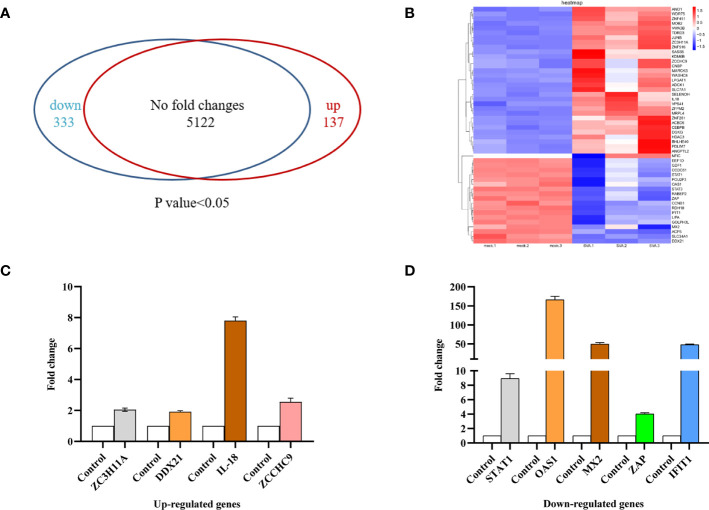
Proteomic analysis of SVA-infected PK-15 cells. PK-15 cells were mock or SVA infected at a MOI of 0.5 and harvested at 48 hpi for proteomic analysis. **(A)** The Venn diagram represents the number of genes upregulated and downregulated in PK-15 cells in response to SVA infection. **(B)** Cluster analysis of the upregulated and downregulated genes with the greatest fold change values. Red indicates increased (infected/mock) gene expression, and blue indicates decreased expression. **(C, D)** Validation of TMT data by qRT-PCR analyses. ZC3H11A, DDX21, IL-18, and ZCCHC9 mRNA levels were upregulated, and STAT1, OAS1, MX2, ZAP, and IFIT1 mRNA levels were downregulated. Values are presented as means ± SD from three independent experiments.

SVA infection affects various host cellular pathways. Knowledge of changes in host protein expression caused by SVA infection helps to understand the interaction between the virus and the host. Our study employed a dimethyl labeling-based quantitative proteomics strategy to characterize the host cell proteome changes upon SVA infection in PK-15 cells at 48 hpi. The results showed that 333 proteins were downregulated and 137 were upregulated in the cells infected with SVA. In contrast, the expression level of 5,122 proteins was not affected by SVA infection **(** **Figure 2A** **)**. These results were also visualized by clustering the samples according to differential treatment **(** **Figure 2B** **)**. To further verify the accuracy of the proteomics and the relationships of the differentially expressed proteins (DEPs) at the translational and transcriptional levels, the transcriptional level of some DEPs (STAT1, IFIT1, OAS1, ZAP, MX2, DDX21, IL-18, ZCCHC9, and ZC3H11A) were verified by qPCR. The results showed that the transcriptional and translational levels are almost identical to DEPs except for DDX21 during SVA infection **(** **Figures 2C, D** **)**. The translational level of DDX21 was downregulated during SVA infection **(** **Figure 2B** **)**. In contrast, the transcription level of DDX21 was upregulated in the SVA-infected cells **(** **Figure 2C** **)**. Thus, the SVA infection inevitably affects the protein function of DDX21.

### DDX21 Restricts the Replication of SVA

**Figure 3:**
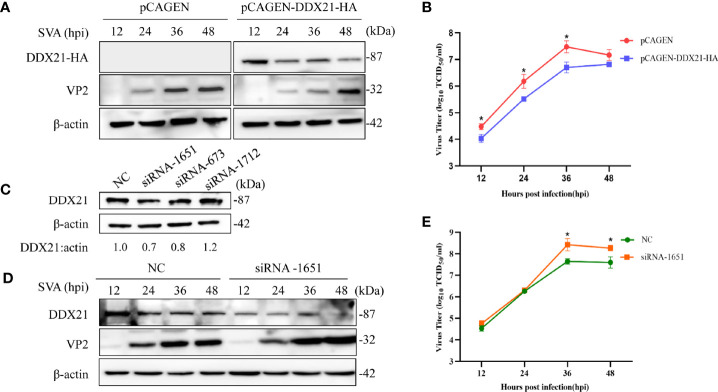
DDX21 affects the replication of SVA. **(A)** BHK-21 cells were transfected with pCAGEN-DDX21-HA or pCAGEN. At 36 hpt, the cells were inoculated with SVA at an MOI of 0.1. The cells and supernatant were harvested at the indicated times, and the expression level of the VP2 protein of SVA was analyzed by WB with an anti-VP2 pAb. DDX21-HA was detected with an anti-HA mAb. β-Actin served as the internal reference. **(B)** Virus titers were calculated and expressed as TCID_50_/ml at the indicated times. **(C)** BHK-21 cells seeded in 6-well plates were transfected with siRNA-1651, siRNA-673, siRNA-1712, or negative control (NC) (50 pM/well). At 36 hpt, the cells were lysed and analyzed by Western blotting (WB) with an anti-DDX21 polyclonal antibody (mAb), and β-actin was probed as the internal reference. **(D)** Knocking down the expression of DDX21 promoted the replication of SVA in BHK-21 cells. BHK-21 cells were transfected with NC or siRNA-1651 at 50 pM. At 36 hpt, the cells were infected with SVA at a MOI of 0.1. The cells and the supernatant were harvested at indicated times. The cells were lysed and analyzed by WB using an anti-VP2 pAb. **(E)** The infectious virus titers in the supernatant were determined by a TCID_50_/ml assay. The statistical significance of differences was determined using a Student’s *t*-test (NS, not significant; ^*^ *p* < 0.05).

To further examine the effects of DDX21 on SVA replication, DDX21 was overexpressed in the BHK-21 cells by transfecting them with pCAGEN-DDX21-HA. As shown in **Figure 3A**, when DDX21 was overexpressed, the VP2 protein levels of SVA were lower than those in the control cells, especially at 12–36 hpi. Furthermore, there was a significant difference in virus titers between cells transfected with pCAGEN-DDX21-HA or pCAGEN, with an approximate 0.5–1.0 log decrease in virus titers from 12 to 36 hpi (*p* < 0.05) **(** **Figure 3B** **)**. By contrast, three specific siRNAs (siRNA-1651, siRNA-673, and siRNA1712) were synthesized to knock down the expression level of DDX21 in BHK-21 cells. The results suggested that siRNA-1651 had the best knockdown effect **(** **Figure 3C** **)**. Thus, siRNA-1651 was used in the subsequent interference experiments. As shown in **Figure 3D**, the expression level of VP2 was increased upon transfection with siRNA-1651, especially 36 and 48 hpi, compared with those in NC-transfected cells. Virus titers in the culture supernatants of cells transfected with siRNA-1651 were also increased, which was consistent with the expression levels of the VP2 protein, with a significant difference at 36 and 48 hpi (*p* < 0.05; **Figure 3E** **)**. These observations suggest that DDX21 is a cellular antiviral factor that represses SVA infection.

### SVA 2B and 3C Degrade DDX21

**Figure 4:**
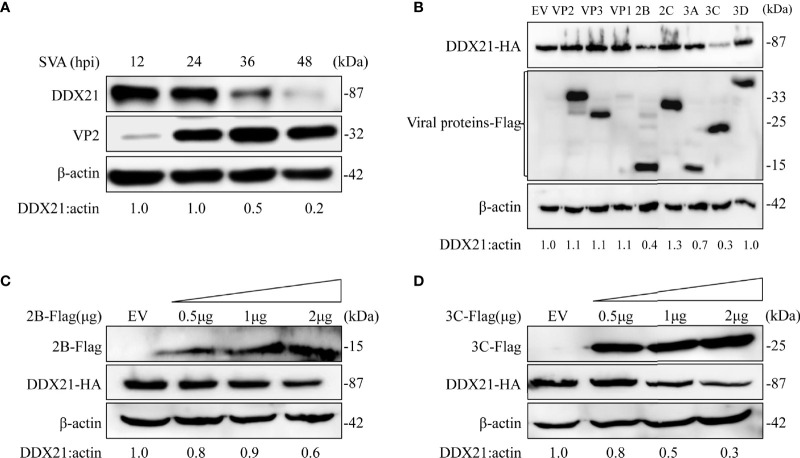
SVA degrades endogenous DDX21 during viral infection, and DDX21 is degraded by SVA 2B and 3C. **(A)** BHK-21 cells in 6-well plates were infected with SVA at a MOI of 0.1, cells were harvested at indicated times, and the expression level of endogenous DDX21 protein was analyzed by WB. **(B)** HEK293T cells in six-well plates were cotransfected with HA-DDX21 and Flag-VP1, VP2, VP3, 2B, 2C, 3A, 3C, or 3D, cells were harvested at 30 h, and the samples were analyzed by WB. **(C, D)** HEK293T cells were cotransfected with HA-DDX21 and different concentrations of Flag-2B or Flag-3C for 30 h. The cell lysates were analyzed by WB.

As shown in **Figure 3A**, the expression level of DDX21-HA decreased gradually upon SVA infection, which was consistent with the proteomics results. Furthermore, the transcription level of DDX21 was upregulated during SVA infection. Therefore, we speculated that SVA infection could degrade DDX21. To verify this hypothesis, BHK-21 cells infected with SVA were lysed at 12, 24, 36, and 48 hpi, and the expression level of DDX21 was analyzed by WB. The results revealed that the expression of DDX21 decreased in SVA-infected cells with the prolongation of infection time **(** **Figure 4A** **)**. Furthermore, to confirm which protein of SVA induces the degradation of DDX21, DDX21 was cotransfected with the protein of SVA. The results revealed that proteins 2B and 3C of SVA induced the degradation of DDX21 **(** **Figure 4B** **)**. Furthermore, the 2B and 3C of SVA degraded DDX21 in a dose-dependent manner **(** **Figures 4C, D** **)**.

### 3C Protein of SVA Degrades DDX21 Depending on Its 3C Protein Protease Activity

**Figure 5:**
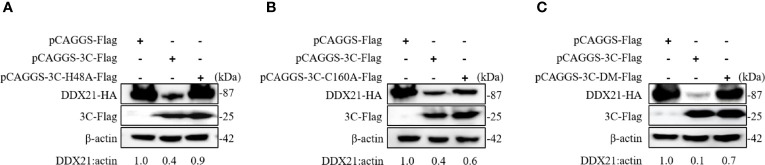
SVA 3C protein targets DDX21 for degradation through its protease activity. **(A)** HEK293T cells were cotransfected with HA-DDX21 and 3C protein or 3C-H48A for 30 h. The cell lysates were prepared for Western blot analysis using the indicated antibodies. **(B)** HEK293T cells were cotransfected with HA-DDX21 and 3C protein or 3C-C160A for 30 h. The cell lysates were prepared for Western blot analysis using the indicated antibodies. **(C)** HEK293T cells were cotransfected with HA-DDX21 and 3C protein or 3C-DM for 30 h. The cell lysates were prepared for Western blot analysis using the indicated antibodies.

The SVA 3C protein contained a conserved catalytic box with histidine (His) and cysteine (Cys) residues, which was essential for its protease activity (15). Single-site mutation H48A (3C-H48A) or C160A (3C-C160A) and double-site mutation H48A-C160A (3C-DM) were catalytically inactive (16). To detect whether 3C protease activity was involved in DDX21 degradation, HEK293T cells were cotransfected with plasmids encoding wild-type 3C protein (3C protein-WT) or its mutants, and HA-DDX21 for 30 h. The results showed that all 3C protein mutants losing protease activity failed to induce DDX21 to degrade **(** **Figures 5A, C** **)**.

### DDX21 Does Not Interact With SVA 2B and 3C Proteins

**Figure 6:**
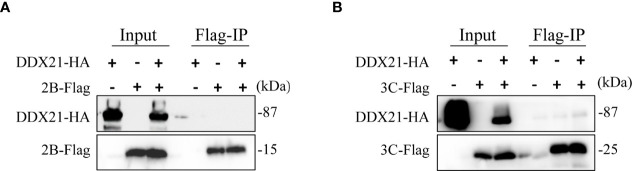
DDX21 does not interact with SVA 2B and 3C protein. **(A)** HEK293T cells were cotransfected with HA-DDX21 and 2B-Flag. **(B)** HEK293T cells were cotransfected with HA-DDX21 and 3C-flag. Coimmunoprecipitation (Co-IP) was performed using agarose beads conjugated with anti-Flag mAb agarose beads. Precipitated proteins were analyzed by WB using antibodies against the Flag and HA.

DDX21 was degraded by SVA 2B and 3C proteins, so we speculate that the 2B and 3C may interact with DDX21. To verify this hypothesis, we performed coimmunoprecipitation (Co-IP) assays to analyze the interaction between nonstructural proteins (2B and 3C) and DDX21. HEK293T cells were cotransfected with plasmids expressing HA-tagged DDX21 and Flag-tagged 2B or 3C proteins. Co-IP assays were performed with anti-Flag monoclonal antibody at 48 hpt. Interestingly, coimmunoprecipitation assays showed that DDX21 did not precipitate 2B and 3C proteins **(** **Figures 6A, B** **)**.

### Effect of Inhibitors on the Viability of Cells

**Figure 7:**
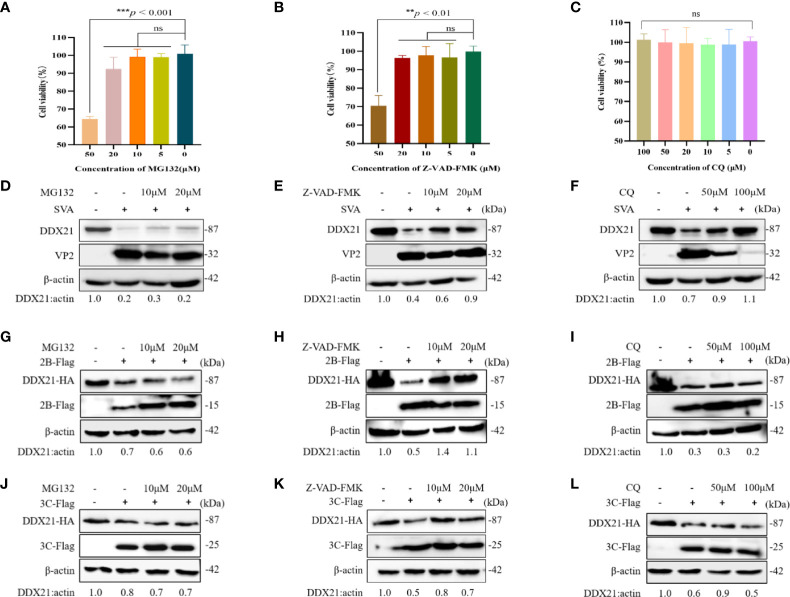
DDX21 is degraded through the caspase pathway during SVA infection. Cells were treated with the indicated concentrations of inhibitors for 24 h. MTT method is used to detect BHK-21 cells cytotoxicity. **(A)** MG132. **(B)** Z-VAD-FMK. **(C)** CQ. **(D–F)** BHK-21 cells in six-well plates were incubated with different inhibitors and then infected with SVA at an MOI of 0.1. The cell lysates were analyzed by WB. MG132 was added at 10 to 20 µM, Z-VAD-FMK was added at 10 to 20 µM, and CQ was added at 50 to 100 µM. **(G–I)** BHK-21 cells were cotransfected with HA-DDX21 and 2B-Flag and then treated with different inhibitors. MG132 was added at 10 to 20 µM, Z-VAD-FMK was added at 10 to 20 µM, and CQ was added at 50 to 100 µM. The cell lysates were analyzed by WB. **(J–L)** BHK-21 cells were cotransfected with HA-DDX21 and 3C-Flag and then treated with different inhibitors. MG132 was added at 10 to 20 µM, Z-VAD-FMK was added at 10 to 20 µM, and CQ was added at 50 to 100 µM. The cell lysates were analyzed by WB. The statistical significance of differences was determined using a Student’s *t*-test (NS, not significant; ^**^ *p* < 0.01; ^***^ *p* < 0.001).

We examined the effects of different inhibitors on the growth of BHK-21 cells at 24 h by an MTT assay. As shown in **Figures 7A, C**, compared with the DMSO-treated group, MG132 (0–20 µM) and Z-VAD-FMK (0–20 µM) did not affect the cell viability. However, a concentration of 50 µM reduced the cell viability. In contrast, CQ (0–100 µM) did not affect cell viability. So, we choose the proteasome inhibitor MG-132 (10–20 µM), the caspase inhibitor Z-VAD-FMK (10–20 µM), and the lysosomal inhibitor CQ (50–100 µM) for subsequent experiments.

### 2B and 3C Protein of SVA Degrade the DDX21 *via* the Caspase Pathway

To further confirm whether DDX21 was degraded upon virus infection, cells were treated with the proteasome inhibitor MG-132, the caspase inhibitor Z-VAD-FMK, and the lysosomal inhibitor CQ, followed by SVA for 36 h. As shown in **Figures 7D–F**, DDX21 degradation was inhibited after treatment with Z-VAD-FMK. Notably, CQ also seemed to inhibit the degradation of DDX21 **(** **Figure 7F** **)**. However, viral protein expression was also inhibited after treatment with CQ, indicating that the inhibition of DDX21 degradation may be due to its severe inhibition of virus replication. Next, we investigated which pathways were involved in the degradation of DDX21 by 2B and 3C proteins. The results showed that 2B and 3C degraded DDX21 through the caspase pathway and not *via* the lysosome and proteasome pathways **(** **Figures 7G–L** **)**. These results suggest that DDX21 was degraded through the caspase pathways.

## Discussions

As an emerging infectious disease pathogen of swine, SVA has demonstrated its capacity to cause vesicular diseases, neonatal mortality, and persistent infection in pigs. Furthermore, it induces immunodepression in suckling piglets and effectively replicates in piglets’ tonsils, mesenteric lymph nodes, and spleen (17, 18). With SVA disease appearing in new countries each year, the virus has become a virus with a global prevalence (19). There must be a molecular mechanism by which SVA antagonizes host antiviral immunity. Although much work has been done in SVA research, there is currently no effective vaccine to control the disease. The interaction of SVA with the host immune response and the pathogenesis remains largely unknown, resulting in the continuous spreading of the disease.

Cell lines have been used as important tools to study virus–host interactions during viral infection. It allows the investigation of the host proteins and pathways involved in viral replication. SVA can infect a variety of cells, such as swine testis (ST), human embryonic kidney-293T, swine kidney (IBRS-2 and PK-15), and human lung cancer cells (NCI-H1299) and BHK-21 (20, 21). To explore the mechanism of SVA infection, the PK-15 cell line can be used as a model for evaluating SVA-induced innate immune signaling and the involved antagonistic effects caused by viral proteins (22). In this study, we screened several candidate cellular proteins in SVA-infected PK-15 by proteomic analysis. In total, 470 DEPs were verified, of which 333 were downregulated and 137 were upregulated. Among the DE proteins, four host proteins with antiviral effects on other viruses were used for screening (10, 23–25). Only DDX21 and IL-18 can suppress the expression of VP2 of SVA (date not shown). Interestingly, unlike IL-18, DDX21 decreased during SVA infection (**Figure 2B**), while the transcription level of DDX21 surprisingly increased **(** **Figure 2C** **)**. Similar phenomena were previously reported for DDX21, DDX23, and DDX1, whose mRNA levels are higher in FMDV-infected cells than in mock-infected cells (11, 26, 27). The transcription level of DDX21 was inconsistent with the translation level, implying that SVA infection affects the function of DDX21. DDX21 is involved in a variety of cell functions, including transcription, protein translation, processing, and modification of pre-rRNA, mediating and sensing transcription during nucleotide stress, as well as regulating various innate immunity (28–31). Many studies have confirmed that DDX21 may act as an antiviral protein. It has been reported that DDX21 restricts infection and replication of several viruses, including the influenza virus, Borna disease virus (BDV), dengue virus (DENV), FMDV, and SARS-CoV-2 (10, 11, 28, 32, 33). To further verify the effect of DDX21 on the virus replication, overexpression and knockdown experiments of DDX21 were conducted. The results demonstrated that DDX21 could effectively inhibit SVA replication in BHK-21 cells **(** **Figures 3A, E** **)**. However, many viruses have evolved general strategies to evade host defenses, such as influencing mRNA translation, protein localization, and cleaving or degrading host proteins (15, 34). In a natural infection, SVA can effectively replicate; therefore, we assumed that SVA could evade the antiviral effect by some means. During the overexpression experiments, we found that as the virus replicated, the expression of DDX21 dropped, which was consistent with the proteomics results. All of these suggest that SVA may escape the antiviral effect of DDX21 by degrading DDX21.

To investigate the mechanism of SVA degrading the DDX21, SVA and the viral proteins were used for verification. With the prolongation of virus infection, the more severe the degradation of DDX21 **(** **Figure 4A** **)**. In addition, only the 2B and 3C proteins of SVA can induce the degradation of DDX21 **(** **Figure 4B** **)**. With the increase of 2B and 3C proteins, the degradation of DDX21 was more significant **(** **Figures 4C, D** **)**. The 2B and 3C proteins are important nonstructural proteins of SVA with multiple roles, especially in antagonizing host innate immunity. 2B protein can suppress type I interferon production by inducing the degradation of MAVS, indicating that 2B protein has the function of degrading and antagonizing innate immunity. It likely enhances membrane permeability, acting like a viroporin (4).

Furthermore, SVA 2AB protein promoted MARCHF8 and MAVS degradation to inhibit IFN-I signaling during SVA infection (35). In contrast, the 3C protein of SVA cleaves PABPC1 to promote viral replication through 3C protease activity (36) and 3C protein-induced hnRNP A1 degradation through its protease activity to promote virus replication (37). SVA suppressed the innate immune response and mediated host cell autophagy and apoptosis to improve viral replication through 2C and 3C proteins (38). Similar to other picornaviruses, the protease activity of the SVA 3C protein depends on a conserved catalytic box with Cys and His residues (16). When the Cys and His residues of 3C protein were mutated (H48A, C160A, or H48A/C160A), it failed to induce the degradation of DDX21, indicating that the degradation of DDX21 by 3C was dependent on protease activity. Since 2B and 3C proteins did not interact with DDX21, the degradation of DDX21 did not depend on their interaction **(** **Figures 6A, B** **)**. Subsequently, we investigated the involvement of the lysosome, proteasome, and caspase pathways in SVA, 2B, and 3C protein-dependent degradation of DDX21. The results revealed that SVA, 2B, and 3C proteins use the caspase pathway to degrade DDX21 **(** **Figure 7** **)**. Although infection with SVA after treatment of cells with CQ can alleviate the degradation of DDX21 **(** **Figure 7F** **)**, it may be related to autophagy. As shown by Hou etal. (39), SVA infection induces autophagy in BHK-21 cells, and SVA utilizes autophagy machinery to promote its proliferation in BHK-21 cells. After treatment with CQ (a specific inhibitor of autophagosome-lysosome fusion), the VP1 protein expression and viral yields were appreciably reduced. Similarly, we obtained the same results as above after CQ treatment. Therefore, we speculated that the inhibition of DDX21 degradation might be due to inhibition of autophagy and decreased VP2 expression in SVA-infected BHK-21 cells. CQ did not alleviate the degradation effects of 2B and 3C proteins on DDX21, so we believe that the lysosomal pathway is not the main way for SVA to degrade DDX21 **(** **Figures 7G–L** **)**.

In summary, we confirmed that DDX21 inhibits SVA replication. Furthermore, SVA can evade the antiviral activity of DDX21 by decreasing its expression through the 2B and 3C proteins of SVA. The degradation of DDX21 by 2B and 3C proteins depends on the caspase pathway. Moreover, SVA 3C protein can degrade DDX21 through 3C protease activity. Our findings provide new insight into the development of antivirals against SVA infection.

## Data Availability Statement

The data presented in the study are deposited in the ProteomeXchange Consortium (http://proteomecentral.proteomexchange.org) repository, accession number PXD034197.

## Author Contributions

KZ designed the experiments. KZ, XRG, SFL, XNL, YH, LLW, BSL, WCZ, and LML performed the experiments. KZ and XRG analyzed the data and wrote the manuscript. WZY made constructive comments on the experiments. All authors contributed to the article and approved the submitted version.

## Funding

This work was supported by the Key Research and Development Projects of Hebei (Grant No. 20326625D); the Talents Introduction Projects of Hebei Agricultural University (Grant No. YJ201945); the State Key Laboratory of Veterinary Etiological Biology, Lanzhou Veterinary Research Institute, Chinese Academy of Agricultural Sciences (Grant No. SKLVEB2020KFKT016); the National Natural Science Foundation of China (Grant No. 32102644); Basic Scientific Research Funds of Provincial Universities in Hebei Province (KY202017); and the Precision Animal Husbandry Discipline Group Construction Project of Hebei Agricultural University (1090064).

## Conflict of Interest

The authors declare that the research was conducted without any commercial or financial relationships that could be construed as a potential conflict of interest.

## Publisher’s Note

All claims expressed in this article are solely those of the authors and do not necessarily represent those of their affiliated organizations, or those of the publisher, the editors and the reviewers. Any product that may be evaluated in this article, or claim that may be made by its manufacturer, is not guaranteed or endorsed by the publisher.
